# Machine Learning of Reaction Properties via Learned Representations of the Condensed Graph of Reaction

**DOI:** 10.1021/acs.jcim.1c00975

**Published:** 2021-11-04

**Authors:** Esther Heid, William H. Green

**Affiliations:** Department of Chemical Engineering, Massachusetts Institute of Technology, Cambridge, Massachusetts 02139, United States

## Abstract

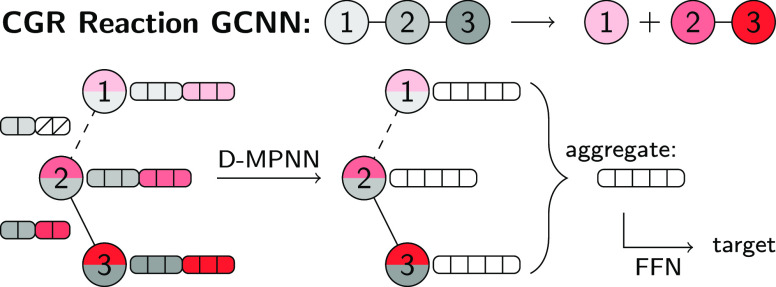

The estimation of chemical reaction properties such as activation energies, rates, or yields is a central topic of computational chemistry. In contrast to molecular properties, where machine learning approaches such as graph convolutional neural networks (GCNNs) have excelled for a wide variety of tasks, no general and transferable adaptations of GCNNs for reactions have been developed yet. We therefore combined a popular cheminformatics reaction representation, the so-called condensed graph of reaction (CGR), with a recent GCNN architecture to arrive at a versatile, robust, and compact deep learning model. The CGR is a superposition of the reactant and product graphs of a chemical reaction and thus an ideal input for graph-based machine learning approaches. The model learns to create a data-driven, task-dependent reaction embedding that does not rely on expert knowledge, similar to current molecular GCNNs. Our approach outperforms current state-of-the-art models in accuracy, is applicable even to imbalanced reactions, and possesses excellent predictive capabilities for diverse target properties, such as activation energies, reaction enthalpies, rate constants, yields, or reaction classes. We furthermore curated a large set of atom-mapped reactions along with their target properties, which can serve as benchmark data sets for future work. All data sets and the developed reaction GCNN model are available online, free of charge, and open source.

## Introduction

Machine learning models to predict molecular properties have seen a large surge in popularity in the past decade, leading to new developments and impressive performances on the prediction of quantum-mechanical properties,^1−3^ biological effects,^4−6^ or physicochemical properties,^7−9^ to name just a few. In particular, graph-based approaches are on the rise and have proven both powerful and useful in fields such as drug discovery.^10^

Many representations and model architectures have been developed for the property prediction of molecules. Popular approaches range from conventional machine learning models on fingerprints or descriptors,^11^ graph-convolutional neural networks on 2D graphs,^1,3,8,9^ and spatial convolutions on 3D coordinates^2,12,13^ to natural language processing on string representations,^14,15^ among others. In contrast, the development of representations and architectures to predict the properties of chemical reactions, i.e., the transformation from one molecule to another, lags behind. Recent studies include the prediction of reaction yields via a random forest model on selected descriptors,^16^ a random forest model on structure-based fingerprints,^17^ or a molecular transformer model on reaction strings.^18^ Reaction barriers were successfully predicted with both linear regression and neural network models on expert-selected features^19^ or Gaussian process regression on selected computational results.^20^ Reaction rates were estimated via deep neural network models on expert features,^21^ as well as selectivities via different models on expert-curated descriptors.^22^ With the notable exception of the seminal work of Schwaller et al.,^18^ all these approaches rely on manually created sets of descriptors or features, which hinders the ability to transfer model architectures and representations to new tasks. Recent advances toward a more data-driven reaction representation mainly concern the field of retrosynthesis,^23−26^ forward reaction prediction,^27−32^ or learning the potential energy surface of a reaction.^33^ Furthermore, a dual graph-convolutional neural network was recently proposed for the prediction of activation energies but is unable to handle imbalanced reactions.^34^ General architectures which can address a variety of reaction properties are still scarce, mainly due to a lack of a general reaction representation.

Within the field of cheminformatics, the condensed graph of reaction (CGR),^35,36^ which is a superposition of the reactant and product molecules of a reaction, was found to be a suitable reaction representation for a diverse set of tasks. It can be easily constructed from an atom-mapped reaction by assigning dual labels to each bond and atom according to their properties in the reactants and products, respectively. A CGR can be computed from both balanced and imbalanced reactions, thus naturally alleviating some of the restrictions of previous reaction representations. Among others, CGRs were successfully used for structure–reactivity modeling,^37−39^ reaction condition prediction,^40,41^ atom-mapping error identification,^42^ and reaction similarity searches.^35^ Toolkits are available to generate or process CGRs, such as the Python library CGRTools.^43^ Despite these promising results, the condensed graph of reaction has not been utilized as input representation to deep learning models, such as graph-convolutional neural networks, yet.

In this study, we therefore adapt a graph-convolutional neural network to encode the condensed graph of reaction instead of a molecular graph and successfully predict reaction properties such as activation energies, reaction enthalpies, rate constants, yields, or reaction classes. The developed architecture is general, versatile, and provides a large improvement in accuracy compared to current reaction prediction approaches over a broad field of tasks.

## Methods

### Condensed Graph of Reaction

**Figure 1:**
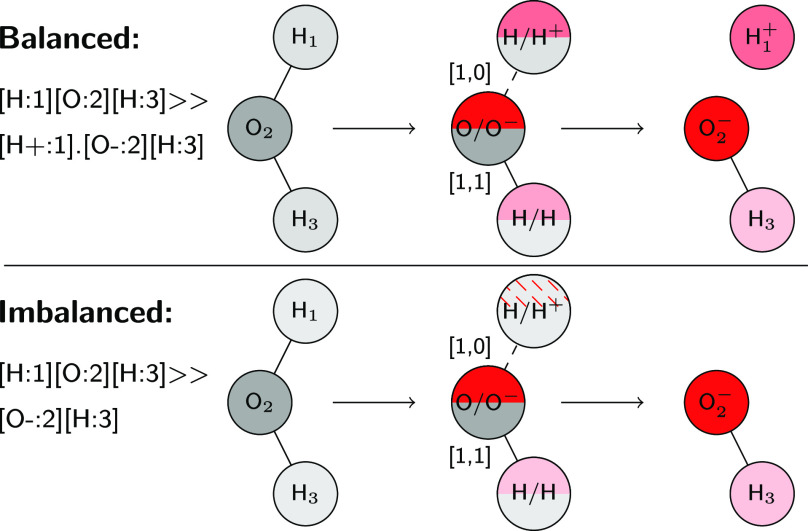
Schematic depiction of the CGR (middle) for the dissociation of water, constructed from the atom-mapped reactants (right) and the atom-mapped products (left). (Top) Example of balanced reaction. (Bottom) Example of imbalanced reaction. In the CGR, each atom and each bond has two labels, one corresponding to the reactants and another to the products. For imbalanced reactions, the features of an imbalanced atom can either be imputed or set to zero (indicated by the striped area).

The CGR is a simple superposition of the reactant and product graphs of the molecules in a reaction. The atom mapping of the reaction links the two graphs and thus provides an important input to correctly construct the CGR. Figure 1 depicts the atom-mapped reactant molecules in gray (left), as well as the atom-mapped product molecule in red (right) for the dissociation of water. In the middle, the resulting CGR is visualized. The two-colored atoms represent the dual properties of each atom before and after the reaction. The bonds undergoing changes are depicted as dashed lines, and the labels indicate the bond type before and after the reaction. Usually, changes in an atom concern its charge, hybridization, multiplicity, or its environment, whereas changes in a bond concern its bond type.^43^ Usually labels that are the same for reactants and products, for example, [1,1] for the bond from O_2_ to H_3_ or H/H for H_3_, are collapsed into a single label,^43^ but we deliberately keep both labels, as each label is used later to construct a part of the atomic and bond features vectors of the CGR graph representation. CGRs can be obtained for both balanced and imbalanced reactions, and imbalanced reactions can be balanced via decomposition of the CGR.^44^ However, correct labels for missing atoms and bonds can only be recovered for some but not all reactions using CGR decomposition, namely, if no rearrangements occurs within the missing fragments. An automatic balancing via the CGR therefore potentially introduces noise to a data set, if some of the missing fragments are wrongly autocompleted. We therefore provide the user with the option to either set the features of the corresponding atoms and bonds to zero, or copy the features from the respective atoms and bonds on the other side of the reaction, to avoid inconsistencies between balanced and imbalanced data sets. The striped area in the bottom part of Figure 1 indicates this choice. Later in this article, the benefit of this treatment over a simple balancing via the CGR is described further.

### D-MPNN Architecture

In the following, we briefly summarize the architecture of molecular-directed message passing neural networks (D-MPNNs), a class of graph-convolutional neural networks (GCNNs), to provide context to the necessary changes and adaptions to generalize from molecules to reactions. We only discuss the directed message passing architecture from ref (8), but the described changes can be easily adapted to any other graph-based architecture.

In general, GCNNs take the graph of a molecule as input, where atoms correspond to vertices in the graph and bonds to edges. The vertices and edges are usually associated with feature vectors, which describe the identity of an atom, as well as the type of a bond. The vertex or edge features are updated iteratively through exchanging information with their neighbors to create a learned representation of each atom. A representation of the whole molecule is then obtained by an aggregation function, often a simple sum or mean of the atomic representations. The molecular embedding is then passed to a readout function, in most cases a feed-forward neural network (FFN) to relate it to a target property. The whole architecture, i.e., the graph convolutions, aggregation, and FFN, are usually trained at the same time, end to end.

**Figure 2:**
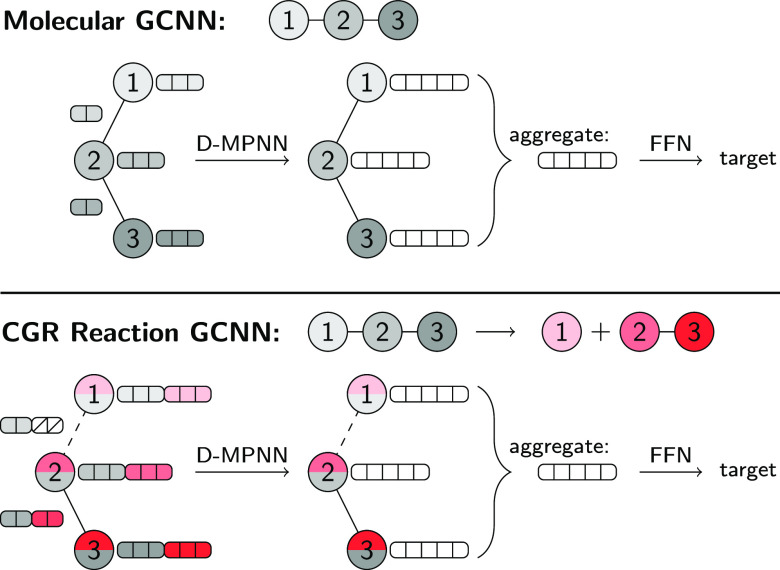
Architecture of a standard graph convolutional neural net (top) and adaption to reactions via input of the condensed graph of reaction (bottom). Each atom and bond fingerprint now consists of two parts, one describing the reactants (gray) and the other the products (red). If a bond does not exist in reactants or products, the corresponding parts of the fingerprint (white, crossed out) are set to zero. If an atom is missing in an imbalanced reactions, its features can be either imputed or set to zero. The white vectors correspond to the hidden atomic and molecular representations.

In the case of D-MPNNs, messages are associated with directed edges instead of vertices, in contrast to regular MPNN architectures. The architecture of Yang et al.^8^ is schematically depicted in Figure 2, top panel. For a molecular graph *G*, initial atom features {*x*_*v*_ |*v* ∈ *V*} for all vertices *V* are constructed from a one-hot encoding of the atomic number, degree, formal charge, chirality, number of hydrogens, hybridization, and aromaticity of the atom, as well as the scaled atomic mass, resulting in vectors of length 133. Initial bond features {*e*_*vw*_ |*vw* ∈ *E*} for all edges *E* describe the bond type, whether the bond is conjugated, in a ring, and contains stereochemical information, resulting in vectors of length 28. The initial directed edge features *h*_*vw*_^0^ are constructed via appending the features of the first atom of a bond, *x*_*v*_ to the respective bond features, *e*_*vw*_, and passing the concatenated vector to a single neural network layer
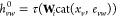
1with  and *h* being the hidden size (default 300), *h*_*i*_ the size of cat(*x*_*v*_,*e*_*vw*_), here 147, and τ()˙ a nonlinear activation function. The directed edge features are then updated via an appointed number of message passing steps *t* = *T* (default 3)
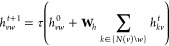
2where  and *N*(v)*w* denotes the neighbors of node *v* excluding *w*. The hidden states are then transformed back to atom features
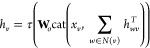
3with  and *h*_*o*_ being the size of *x*_*v*_ and *h*. The atomic representations *h*_*v*_ can then be aggregated to a molecular feature vector
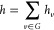
4and optionally augmented with precomputed molecular features *f* as cat(*h*,*f*). The molecular fingerprints are then passed to one or multiple FFN layers.

To adapt the D-MPNN architecture to reactions, two main changes are necessary. First, the list of bonds now encompasses all pairs of atoms that have a bond in either the reactants or the products or both, i.e., *E* = *E*^reac^ ∪ *E*^prod^ of the reactant *G*^reac^ and product *G*^prod^ graphs. Likewise, the list of atoms comprises all atoms that are present in either reactants, products, or both, *V* = *V*^reac^ ∪ *V*^prod^. Second, the initial atom and bond feature vectors now contain two parts, extracted from the reactant and product graphs separately, one corresponding to the reactants and the other to the products or the difference between reactants and products. If an atom or bond only occurs on one side of the reaction, the user is provided with a choice to either set its respective feature vector to zero on the other side or to directly copy over its features to the other side for all atoms and bonds unless a bond is broken within the reaction. Some of the copied features can be incorrect, especially for atoms close to the reactive center, but the reliability of the imputed data can be learned by comparing the features with the structure of the graph. For example, an unbalanced atom next to a broken bond will have a wrong degree (number of neighbors) copied over from the other side of the reactant, which can be identified by comparing against the actual number of neighbors in the graph. If not indicated otherwise, we follow the first approach (setting features to zero) in the remainder of the article but found the performance of both options to be equal on data set 8. We do not provide an option for automatic balancing via the CGR since some imbalanced reactions cannot be autocompleted correctly due to possible rearrangements in the missing fragments, introducing noise to a data set and therefore decreasing model performance (tested on data set 8, data not shown). For atoms, we do not repeat the one-hot encoding of the atomic number, since it cannot change during a chemical reaction, but the scaled mass information is kept for both reactants and products to not lose isotope information in case of imbalanced reactions. We tested different combinations of the reactant and product features to yield the CGR features, namely, to concatenate the reactant and product features directly, to concatenate the product features with the difference between reactant and product features, and to concatenate the reactant features with the difference between product and reactant features, and found that the last option (reactant + difference) usually performs best. All results reported in this study were obtained with this setting, i.e., *x*_*v*_ = cat(*x*_*v*_^reac^, *x̃*_*v*_^diff^) with length 165, where the tilde denotes the vector missing the atomic number information, and *e*_*vw*_ = cat(*e*_*vw*_^reac^,*e*_*vw*_^diff^) with length 28. All options are available in the provided code on GitHub^45^ and can be tuned as hyperparameters. The bottom panel of Figure 2 schematically depicts the adapted architecture, where the gray parts of the initial fingerprints correspond to the reactants and the red parts to the products. The two changes thus only concern the creation of the graph object, as well as the initialization of the edge and vertex features. The remaining parts of the model, i.e., eqs 1–4, are unchanged.

### Data Preparation

We utilized four reaction databases from the literature as provided, as well as cleaned and atom mapped four more, which we made openly available on GitHub.^53^Table 1 provides a compact overview over all employed data sets.

(1) Computational activation energies of forward and reverse reactions at the ωB97X-D3/def2-TZVP level of theory (as well as at the B97-D3/def2-mSVP level of theory for pretraining) were used as provided in ref (46). The data set features a diverse set of reactions transforming unimolecular reactants into unimolecular or multimolecular products and is already atom mapped. All reactions were balanced and contained explicit hydrogens.
(2) Computational activation energies for competing E2/S_*N*_2 were taken from ref (47) and atom mapped manually using heuristic substitution patterns. The resulting database is published along with this study. All reactions were balanced and contained explicit hydrogens.
(3) Experimental activation energies for S_N_Ar reactions were taken as provided from ref (20). All reactions were already atom mapped and furthermore contained information about the solvent each reaction was carried out in, as well as the computational activation energy at the ωB97X-D/6-311+G(d,p) level of theory. The solvent descriptors (vectors of length 5) and computational activation energies (single value) were passed to the model as molecular fingerprints *f* as provided from ref (20). All reactions were balanced and contained implicit hydrogens only.
(4) Computational reaction enthalpies were taken from the Rad-6-RE database^48^ and atom mapped via Grzybowski’s algorithm.^54^ Imbalanced reactions (less than 2% of the data) were discarded, since ref (48) explicitly claims to only report balanced reactions. We thus assumed that imbalanced reactions correspond to errors. Both forward and reverse reactions were taken into account. All resulting reactions were balanced and contained explicit hydrogens. The resulting database is published along with this study. We note that reaction enthalpies could also be modeled via training a single model to predict molecular enthalpies^55,56^ and converting the enthalpies of reactants and products into the respective enthalpies of reaction. This approach was followed by Stocker et al.;^48^ however, in this work, we instead want to highlight the direct prediction of reaction enthalpies.
(5) Reaction rate constants were taken from ref (49) and atom mapped via Grzybowski’s algorithm.^54^ Models were then trained on the logarithm of the rate constants at 1000K, , with *k* in cm^3^ mol^–1^s^–1^ (bimolecular) or s^–1^ (unimolecular) depending on the reaction mechanism, and *k*_*ref*_ = 1 in the same units. The resulting database is published along with this study. All reactions were balanced and contained explicit hydrogens.
(6) Experimental reaction yields for 218 phosphatase enzyme sequences on 157 substrates were extracted from ref (50). The original article features 167 substrates, but only substrates that contained a single phosphate group were kept. Since the reaction outcomes were not reported in ref (50), the products for multiphosphate substrates are not known with certainty and were thus not included. The different enzymes were represented as simple one-hot encoding and passed to the model as molecular fingerprints *f*. Products and the respective atom mappings were calculated manually with a simple set of heuristic rules. The resulting database is published along with this study. All reactions were balanced and contained implicit hydrogens only.
(7) The reaction names of one million reactions from an in-house preprocessed and cleaned version of Pistachio^51^ (processing analogous to ref (57)) were taken with atom mappings as provided. Since Pistachio is not open source, the resulting database is not published along with this study. The reactions were imbalanced, missing leaving groups on the product side, and contained implicit hydrogens only.
(8) The reaction names of the atom-mapped USPTO-1k-TPL data set recently curated by Schwaller et al.^52^ were used as is. The reactions were imbalanced, missing leaving groups on the product side, and contained implicit hydrogens only.

**Table 1:** Summary of Employed Data Sets (a)

data set	data points	ref	H	bal.	split	task	span	MAE	RMSE	unit	epochs
*E*_*a*_ ωB97X-D3^b^	23,923	(46)	yes	yes	dir. scaffold	regression	0 to 205	25.1 ± 0.0	31.0 ± 0.0	kcal/mol	100
*E*_*a*_ E2/S_*N*_2	3626	(47)	yes	yes	random	regression	0 to 65	11.0 ± 0.4	13.3 ± 0.5	kcal/mol	100
*E*_*a*_ S_*N*_Ar	443	(20)	no	yes	random+^c^	regression	13 to 42	2.7 ± 0.4	3.6 ± 0.6	kcal/mol	500
Δ*H* Rad-6-RE	63,849	(48)	yes	yes	dir. scaffold	regression	–6 to 12	3.4 ± 0.0	3.9 ± 0.0	eV	100
log(*k*) rate const.	779	(49)	yes	yes	random	regression	–5 to 10	1.9 ± 0.1	2.2 ± 0.1	unitless	100
Yield phosphatases	33,355	(50)	no	yes	random+^c^	regression	0 to 1^d^	0.10 ± 0.01	0.14 ± 0.01	unitless	100
Pistachio	1,074,765	(51)	no	no	random	multiclass	937^e^	–	–	–	30
USPTO-1k-TPL	445,117	(52)	no	no	predefined	multiclass	1000^e^	–	–	–	30

a Use of explicit hydrogens, whether reactions are balanced, type of split and task, span of targets, performance of dummy model evaluated on five folds, units and number of epochs.

b Pretraining on 32,731 data points at the B97-D3 level of theory.

c Random splits ensuring that identical reactions with different additional features (solvents or enzymes) are put in the same set.

d Four data points have yields higher than 1 due to uncertainties in the assay evaluation.

e Number of classes.

### Dummy Baselines

The mean absolute error of a dummy baseline model predicting the mean of the training target values for all test reactions in each data set is given in Table 1, averaged over five folds. Comparing against such a simple baseline helps to judge the quality of a predictive model, where low errors on a data set with narrow target range can otherwise be mistaken for a satisfactory performance.

### Other Baselines

We furthermore examined more complex baseline models. First, the dual GCNN model of Grambow et al.^34^ was trained with hyperparameters similar to the CGR GCNN approach (MPNN depth of 3, hidden size of 300, one FFN layer, no dropout) on all data sets comprising balanced reactions, termed “Grambow” in the following. The model computes atom embeddings of all atoms in the reactant and product molecules for the reactants and products separately via directed message passing and then subtracts the reactant from the product atom embeddings before aggregating the atomic to molecular embeddings and passing them to a FFN. We note that the model does not accept imbalanced reactions as input, so that no baseline could be computed for the imbalanced data sets (sets 7 and 8) in Table 1.

Second, the recently developed BERT deep learning reaction fingerprints^52^ were utilized as input to a regular FFN, where we used a default hidden size of 300 and two FFN layers. The fingerprints were computed using the open-access rxnfp software on nonatom-mapped reaction SMILES.^58^ BERT reaction fingerprints are vectors of size 256 obtained from a pretrained transformer-based model trained on the classification of nonannotated, text-based representations of chemical reactions.

Third, Morgan fingerprints^59^ were calculated for the reactants and products separately and either subtracted (“Morgan Diff”) or concatenated (“Morgan Concat”) and again served as input to an FFN of hidden size 300 and two FFN layers. Morgan fingerprints at radius 3 and length 1024 were calculated via RDKit.^60^

Fourth, we utilized ISIDA descriptors^35,43^ as inputs to an FFN of hidden size 300 and two FFN layers (sequential fragment features calculated on the CGRs, maximum fragment length of 4). ISIDA descriptors are count vectors of all CGR fragments of a certain size in the data set. Their length depends on the number of distinct fragments in a data set and ranges up to several tens of thousands for the large and diverse data sets 1 and 4.

### Model Parameters

A hyperparameter search for the optimal hidden size, number of layers, number of message passing steps, and dropout rate was computed via 20 steps of Bayesian optimization for the CGR GCNN, Grambow’s dual GCNN, and all fingerprint models as implemented in Chemprop.^8^ Optimized models are termed “opt” throughout this study. More details are given in the Supporting Information. All models were trained with a batch size of 50, ReLU activation functions, mean aggregation between the MPNN and FFN step, and explicit hydrogens as specified in Table 1. Learning rates were increased linearly from 10^–4^ to 10^–3^ for two epochs and then decreased exponentially from 10^–3^ to 10^–4^. Prior to hyperparameter optimization, no dropout, three iterations of message passing, and a hidden size of 300 were used (termed “default”). Regression models used mean absolute error as the metric for evaluation and early stopping; classification models instead used accuracy as the metric. All models were trained on five different data splits to arrive at a split-independent estimate of the true model performance. Split sizes of 80/10/10 for training, validation, and test sets were used if not indicated otherwise. Table 1 lists the split types for each data set. Scaffold splits were performed on the reactant side of the *E*_*a*_ ωB97X-D3 and Δ*H* Rad-6-RE databases, where multiple molecular scaffolds were identified. Both the *E*_*a*_ ωB97X-D3 and the Δ*H* Rad-6-RE data sets comprise forward and reverse reactions, so that special care was taken to enforce that each pair of forward and reverse reactions was placed in the same set (indicated by “dir. scaffold” in Table 1). Otherwise, the test set error of a model is unrealistically low and does not reflect the true model performance. The *E*_*a*_ S_*N*_Ar and yield phosphatases data sets contained identical reactions at different conditions (solvents or enzymes), so that a random split on unique reactions was performed to ensure that identical reactions were placed in the same set (indicated by “random+” in Table 1). Random splits were performed on the remaining data sets (*E*_*a*_ E2/S_*N*_2 and log(*k*) rate constants) since they consisted of too few scaffolds to perform a meaningful scaffold split. A random split was furthermore performed on the Pistachio data set. For the USPTO-1k-TPL data set, the split into training and test data was taken from ref (52), and the training set was split into training and validation sets randomly.

## Results and Discussion

Table 2 summarizes the performances of the CGR GCNN developed in this study, Grambow’s dual GCNN,^34^ and the best performing fingerprint model (FFN on either the Bert, ISIDA, Morgan Diff, or Morgan Concat fingerprints). A full list of test performance (MAE, RMSE, and R^2^ scores) of all default and optimized models on all tasks is available in the Supporting Information. The CGR approach outperforms all other models both with its default hyperparameters, as well as after hyperparameter optimization for all data sets. We also attempted to make comparisons to the reaction data presented in ref (46) (Δ*H* Rad-6-RE), but for technical reasons discussed in the SI, it is difficult to fairly compare the methods on this particular data set. In all systems, the default hyperparameters are close to the ideal hyperparameters, indicating that even the small, compact default model is able to learn complex target properties. In the following, we analyze the performances on each target in detail.

**Table 2:** Comparison of Performances and Respective Number of Trainable Parameters of Regression Tasks between the CGR Graph Convolutional Model of This Study, Grambow’s Dual GCNN of Ref (34) and the Best Performing FFN on Reaction Fingerprints^a^

Data set	unit	CGR default	CGR opt	Grambow default	Grambow opt	best FP opt
**Model performance MAE**						
*E*_*a*_ ωB97X-D3 (pretr. B97-D3)	kcal/mol	4.84 ± 0.29	**4.25** ± **0.19**	6.35 ± 0.26	5.26 ± 0.15	7.55 ± 0.48
*E*_*a*_ E2/S_*N*_2	kcal/mol	**2.64** ± **0.10**	2.65 ± 0.09	2.76 ± 0.08	2.86 ± 0.07	3.00 ± 0.10
*E*_*a*_ S_*N*_Ar	kcal/mol	**0.85** ± **0.12**	0.91 ± 0.11	1.04 ± 0.17	0.94 ± 0.21	0.98 ± 0.13
Δ*H* Rad-6-RE	eV	0.16 ± 0.01	**0.13** ± **0.01**	0.40 ± 0.01	**0.08 to 0.43**^b^	0.65 ± 0.01
log(*k*) rate constants	unitless	**0.41** ± **0.05**	0.41 ± 0.02	0.60 ± 0.05	0.45 ± 0.04	0.59 ± 0.06
Yield phosphatases	unitless	**0.062** ± **0.005**	0.063 ± 0.006	0.077 ± 0.004	0.066 ± 0.007	0.066 ± 0.007
						
**Model performance RMSE**						
*E*_*a*_ ωB97X-D3 (pretr. B97-D3)	kcal/mol	7.63 ± 0.43	**6.88** ± **0.38**	9.11 ± 0.51	7.98 ± 0.37	11.80 ± 0.78
*E*_*a*_ E2/S_*N*_2	kcal/mol	**3.59** ± **0.09**	3.61 ± 0.07	3.74 ± 0.13	3.83 ± 0.11	4.10 ± 0.17
*E*_*a*_ S_*N*_Ar	kcal/mol	**1.22** ± **0.16**	1.25 ± 0.14	1.46 ± 0.23	1.36 ± 0.26	1.43 ± 0.20
Δ*H* Rad-6-RE	eV	0.28 ± 0.02	**0.25** ± **0.02**	0.55 ± 0.03	**0.14 to 0.56**^b^	0.88 ± 0.02
log(*k*) rate constants	unitless	**0.66** ± **0.29**	0.66 ± 0.24	1.00 ± 0.14	0.76 ± 0.26	1.03 ± 0.08
Yield phosphatases	unitless	**0.103** ± **0.007**	0.103 ± 0.008	0.115 ± 0.006	0.108 ± 0.010	0.107 ± 0.010
						
**Model size:**						
*E*_*a*_ ωB97X-D3 (pretr. B97-D3)		378,601	10,371,601	361,801	24,877,601	72,747,201
*E*_*a*_ E2/S_*N*_2		378,601	2,817,101	361,801	16,754,401	334,801
*E*_*a*_ S_*N*_Ar		380,401	1,661,401	361,807	8,278,201	6,396,001
Δ*H* Rad-6-RE		378,601	10,371,601	361,801	20,035,401	6,381,601
log(*k*) reaction rates		378,601	6,393,701	361,801	8,269,801	7,363,201
Yield phosphatases		444,001	6,692,001	362,019	6,390,001	6,387,101

a Intervals correspond to the mean and standard deviation of five folds. Best performance per data set is highlighted in bold.

b See SI for details on the Rad-6-RE model performance.

### Prediction of Activation Energies

**Figure 3:**
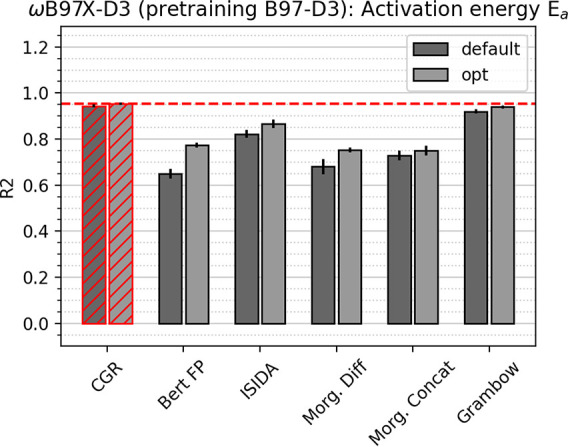
Comparison of test set R^2^ scores between different models for the ωB97X-D3 computational activation energy data set with pretraining on B97-D3 activation energies. Error bars correspond to the standard deviation between five folds. Best model system highlighted in red; line corresponds to best performance.

The performance of the CGR model for the prediction of computational and experimental activation energies was evaluated on three different data sets. The first data set, *E*_*a*_ ωB97X-D3, is by far the largest and most diverse data set, comprising about 24,000 computational activation energies for various elemental reactions in the forward and reverse direction. Its wide range of target values (0–205 kcal/mol) makes an accurate prediction extremely challenging, so that we consider the observed lowest errors of about 4 kcal/mol a success nevertheless. The corresponding high R^2^ score of 0.94 validates this observation. For comparison, a model predicting the mean of the data set for each data point would possess a mean absolute error of about 25 kcal/mol and an R^2^ score of 0. Figure 3 depicts the performance measured via the R^2^ score (with values closest to 1 indicating best performance) of various default and optimized architectures, where the CGR model clearly outperforms other models. Analogous figures with the MAE and RMSE are shown in the Supporting Information. All fingerprint models perform rather poorly, highlighting the inability of reaction fingerprints to encode certain details of a transformation especially for diverse data sets, even despite the large sizes of some of the optimized models. We furthermore note that the obtained performance of the dual GCNN model differs from the results in ref (34) due to the different, more rigorous data splits used in this study. As mentioned in the previous section, placing forward and reverse reactions in different data splits, so that some of the reactions in the test set also appear in the training set (but in reverse direction), can severely overestimate model performance. The errors reported in Table 2 and Figure 3 thus provide a more accurate estimation of the true predictive power of Grambow’s dual GCNN model than the numbers reported in ref (34).

**Figure 4:**
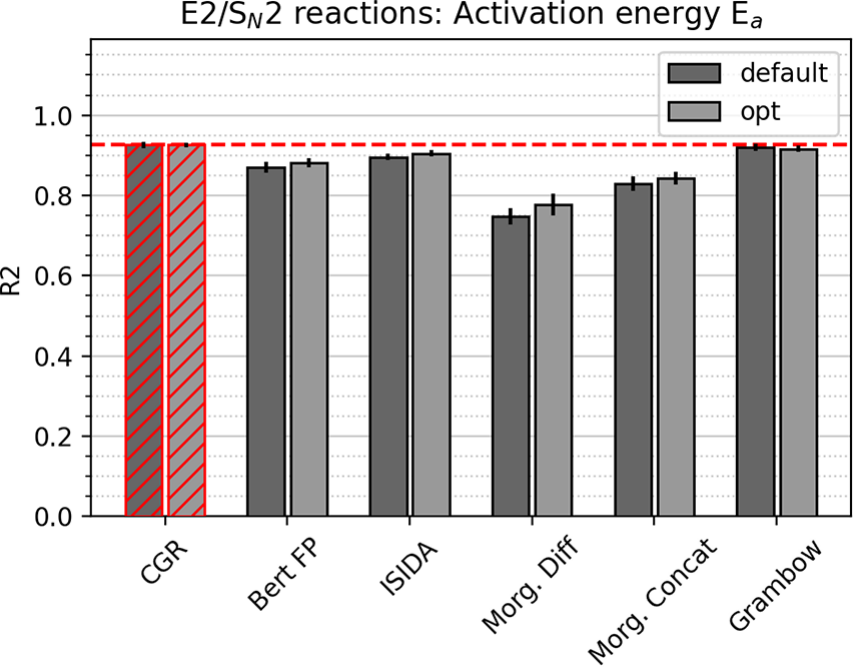
Comparison of test set R^2^ scores between different models for the E2/S_*N*_2 computational activation energy data set. Error bars correspond to the standard deviation between five folds. Best model system highlighted in red; line corresponds to best performance.

The second data set, *E*_*a*_ E2/S_*N*_2, only comprises two chemical transformations, namely, E2 and S_*N*_2 of different electrophiles and nucleophiles. It spans computational activation energies of 0–65 kcal/mol and possesses only a few thousand data points. The baseline performance of a model predicting the mean of the data set for each data point is about 11 kcal/mol. This reduction in target range and chemistry helps all models to perform better regarding RMSE and MAE, but also regarding the *R*^2^ scores, as depicted in Figure 4. Again, the CGR approach outperforms all other models but by a smaller margin. Also, the fingerprint models feature a comparatively better performance than with the previous data set, since the possible chemical transformations are very few, and differences in the activation energies can be related to the fingerprints of reactants and products more straightforwardly.

**Figure 5:**
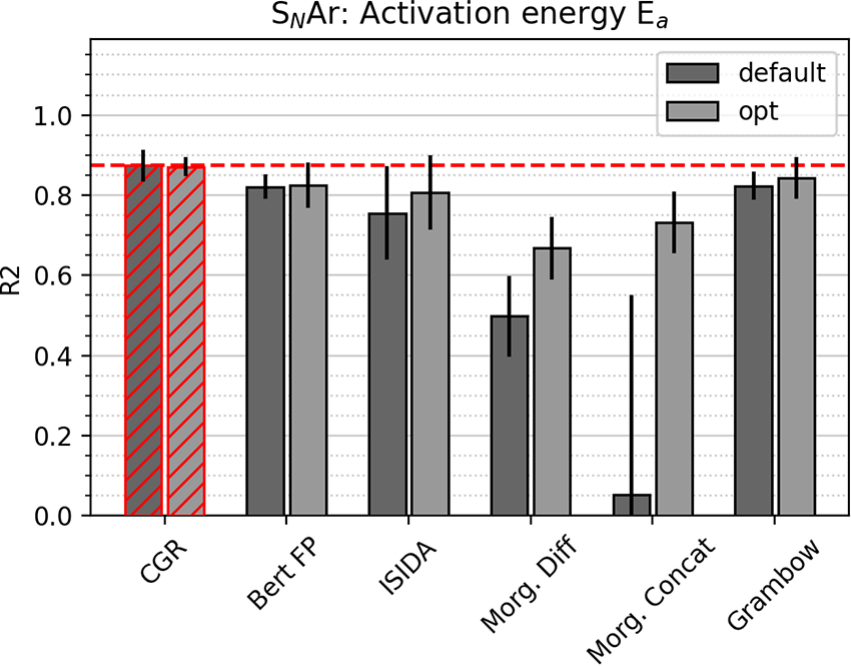
Comparison of test set R^2^ scores between different models for the S_*N*_Ar experimental activation energy data set. Error bars correspond to the standard deviation between five folds. Best model system highlighted in red; line corresponds to best performance.

The third data set, *E*_*a*_ S_*N*_Ar, is different from the first two data sets in three regards. First, it is very small, comprising only a few hundred reactions. Second, it is very narrow, spanning only values between 13 and 42 kcal/mol, which enables even a simple baseline model predicting only the mean of the distribution to perform, seemingly, well with a mean absolute error of about 3 kcal/mol. Third, additional input beyond the reaction itself is provided, namely, five solvent descriptors to characterize the employed solvent and the computational activation energy. Figure 5 depicts the performance of all studied models as measured by the *R*^2^ score, where the CGR approach leads to highest scores but is not significantly better than the optimized Grambow dual GCNN model. In the literature, Gaussian process regression on a large set of quantum-mechanically derived descriptors for this data set yielded a mean absolute error of 0.77 kcal/mol.^20^ The CGR GCNN approach comes reasonably close to this benchmark (MAE of 0.85 kcal/mol, R^2^ of 0.93), taking into account that it only learns from the reaction graphs and does not feature any quantum-mechanical descriptors apart from the solvent information and the computed Ea’s. The requirement for quantum-mechanical descriptors as input can greatly increase the computer time required to make a prediction, but it may be possible to avoid this by building a model for predicting the quantum-mechanical descriptors as was done recently by Guan et al.^61^

**Figure 6:**
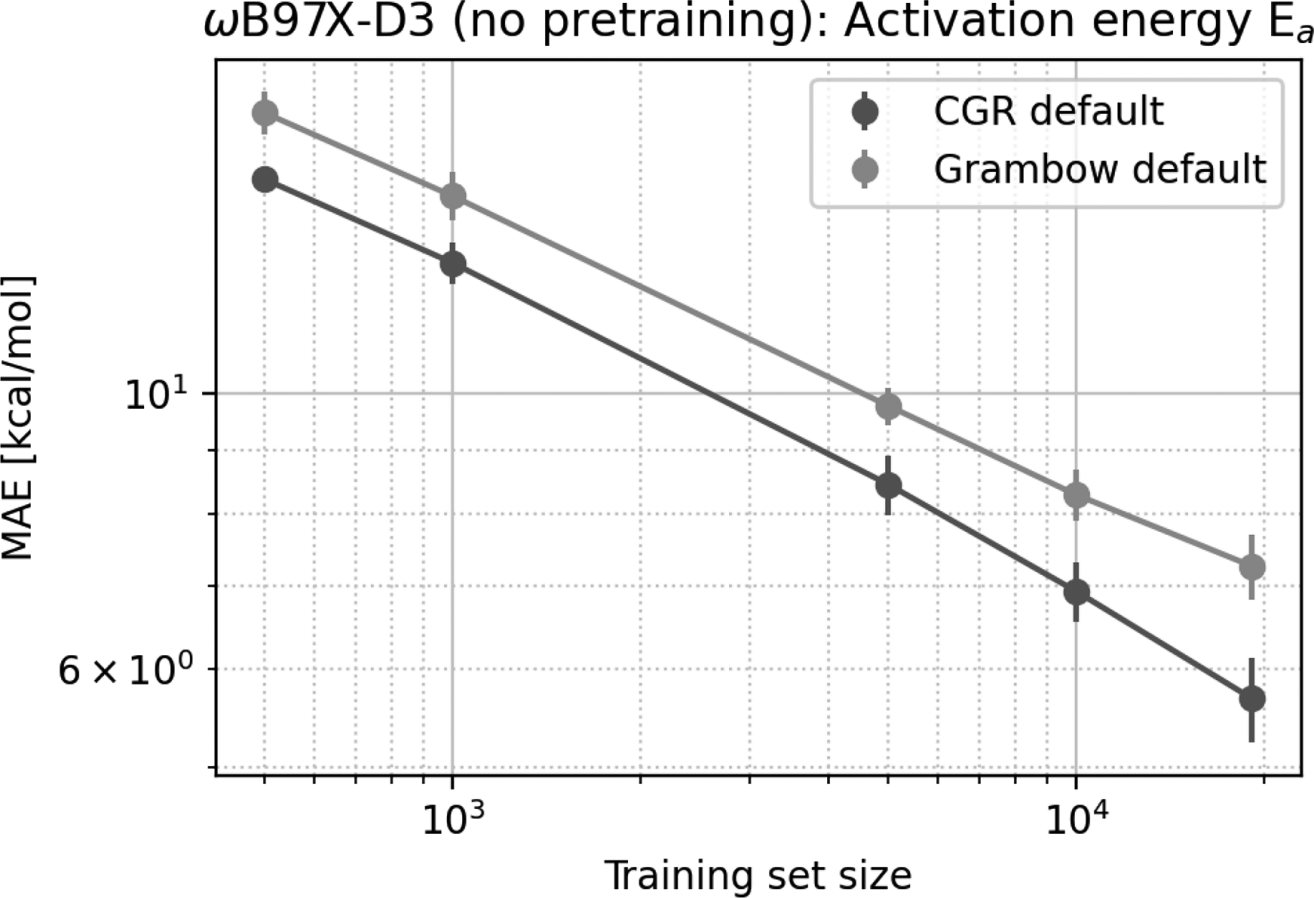
Mean absolute errors of the CGR GCNN model on subsets of the *E*_*a*_ ωB97X-D3 data set without pretraining.

A comparison of the performance of the CGR architecture to the dummy baselines across the three data sets yields another interesting insight. Even with very little data (*E*_*a*_ S_*N*_Ar), the CGR model can still produce a relatively low MAE, at approximately a third of the error of the dummy model. Adding more data, the MAE decreases to a fourth of the dummy model MAE (*E*_*a*_ E2/S_*N*_2), or even a sixth (*E*_*a*_ ωB97X-D3), with further reduction expected for more data points. An evaluation of model performance with training set size for the *E*_*a*_ ωB97X-D3 data set without pretraining is shown in Figure 6 for the default CGR and dual GCNN models. The CGR GCNN model performance does not level off, indicating that the model may achieve chemical accuracy if a sufficiently large data set was provided. A simple extrapolation predicts the model to achieve chemical accuracy with 5–10 million data points, which is not out of reach in light of the current advances in high-performance computing. In contrast, the dual GCNN model levels off slightly, and even if linear behavior is assumed, it would only reach chemical accuracy at 100–300 million data points.

### Prediction of Rate Constants

**Figure 7:**
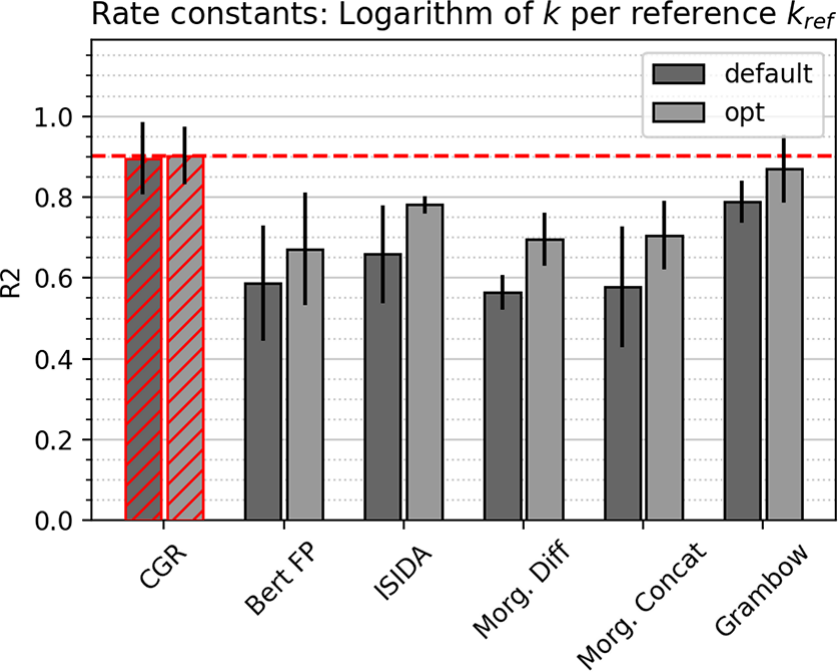
Comparison of test set R^2^ scores between different models for the computational rate constants data set. Error bars correspond to the standard deviation between five folds. Best model system highlighted in red; line corresponds to best performance.

R^2^ scores for predicting rate constants (at 1000K) are shown in Figure 7, where again the CGR GCNN outperforms other approaches with an R^2^ score of 0.90 and an MAE of 0.41 kcal/mol. We note that the errors are reported for the logarithm of the rate constant, so that an MAE of 0.4 corresponds to deviations of about 2.5 in units of cm^3^ mol^–1^s^–1^ (bimolecular) or s^–1^ (unimolecular). This is well within or even below the accuracy of the rates at the employed level of theory, M06-2X/MG3S (compared to more elaborate computational results utilizing CCSD(T)-F12/RI calculations with the cc-VTZ-F1256 and cc-VTZ-F12-CABS57 basis sets, see ref (49)).

### Prediction of Reaction Yields

**Figure 8:**
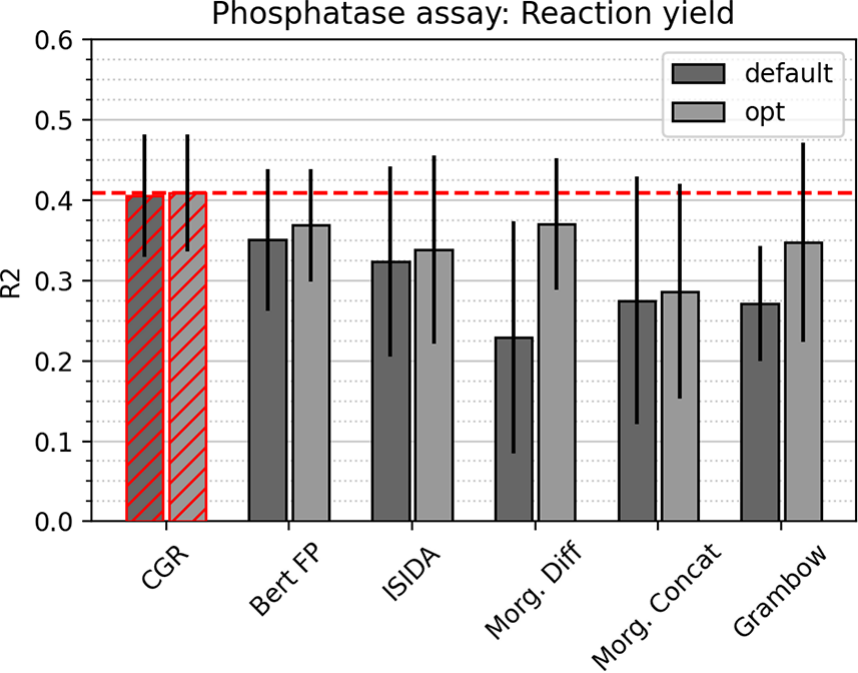
Comparison of test set R^2^ scores between different models for the experimental phosphatase reaction yield data set. Error bars correspond to the standard deviation between five folds. Best model system highlighted in red; line corresponds to best performance.

A different picture arises for the prediction of reaction yields (Figure 8). All models perform about equally well and are only slightly better than a dummy baseline model (with an R^2^ of 0) predicting the mean of the distribution. The CGR approach outperforms other models by a slight, nonsignificant margin, but overall, all model performances are rather mediocre. Since the data set contains only 157 substrates in combination with 218 enzymes, and the enzymes were merely one-hot-encoded, the subprime performance is not surprising. In other words, the models can pick up relations for the different substrates well but is hampered by the crude encoding of the protein information.

### Prediction of Reaction Classes

**Figure 9:**
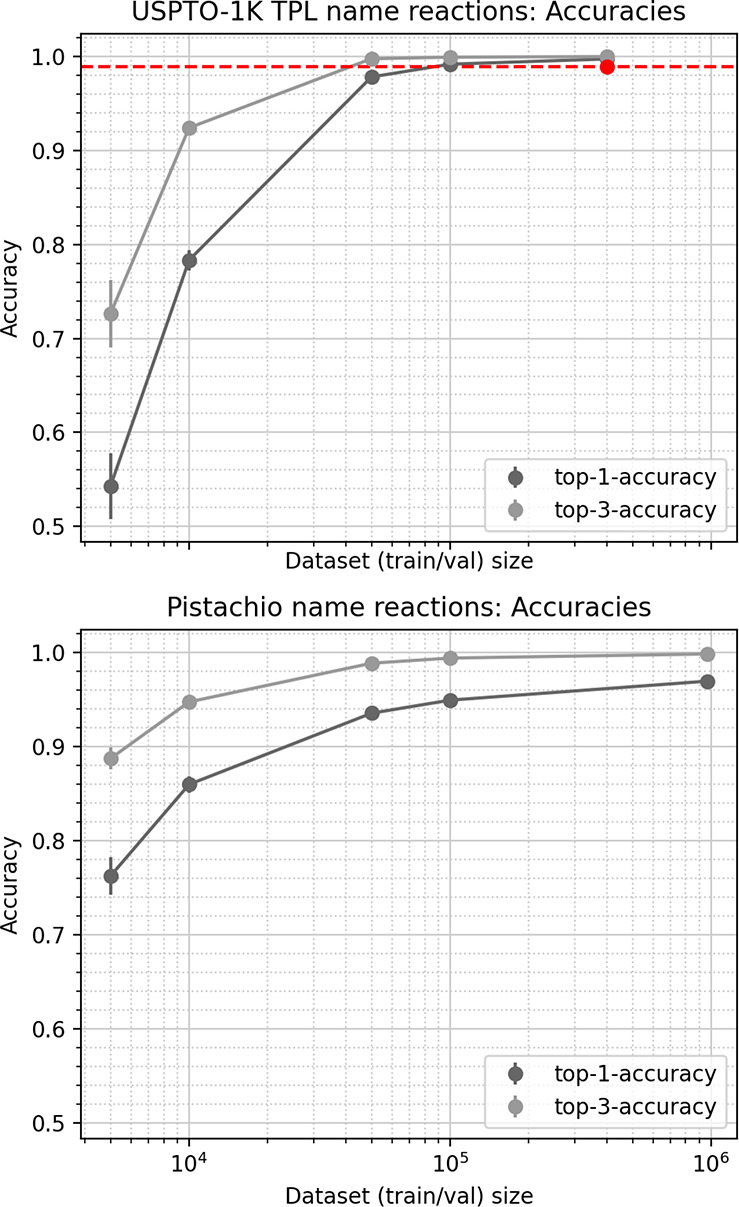
Comparison of accuracies between different models for the classification of name reactions via the USPTO-1K-TPL data set (top) or the Pistachio data set (bottom). Error bars correspond to the standard deviation between five folds. The red dot and line correspond to the performance achieved by ref (52).

We furthermore explored the performance of the CGR GCNN approach on classification tasks, here the classification of reactions into their respective name reactions. To this aim, we predict the names of reactions of two data sets, a preprocessed and cleaned version of Pistachio containing 937 class names, as well as a recently published benchmark, USPTO-1k-TPL, containing 1000 class names. Figure 9 depicts the top-1 accuracy (fraction of test reactions where the correct name is ranked highest) and top-3 accuracy (fraction of test reactions where the correct name is found within the three highest ranked predictions), depending on the size of the training set. Since the reactions in both data sets are not balanced (leaving groups are not reported on the product side), the performance of Grambow’s dual GCNN approach could not be evaluated. We instead compare the observed accuracy to a recent benchmark of Schwaller et al. (red line in Figure 9), who achieved a 98.9% top-1 accuracy on USPTO 1k TPL with their state-of-the-art transformer model.^52^ They furthermore report 98.2% accuracy on Pistachio name reactions but preprocessed and cleaned the data differently, so that no direct comparison is possible. We note that the reaction input to the transformer model does not rely on atom mapping, so that the model learns from less information. The CGR approach outperforms the transformer model, but due to the differences in representation (no atom mapping vs atom mapping), a direct comparison is somewhat biased. Nevertheless, the observed accuracies of the CGR GCNN model indicate that it can learn to predict name reactions easily and that imbalanced reactions do not hamper model training.

### Limitations

The CGR GCNN approach developed in this study thus provides a high-performing and flexible alternative to other architectures, such as dual GCNN and FFNs on various fingerprints. It is more flexible than the dual GCNN model in that it can treat imbalanced reactions. However, like the dual GCNN architecture, it relies on correct atom mapping of reactions, which increases the work load on preprocessing steps of databases significantly. Incorrect atom mappings add noise to the data, so that the quality of a prediction depends to some extent on the quality of the atom mapping of both training and test data.

## Conclusions

We have introduced, benchmarked, and validated the use of CGRs as a suitable reaction representation to graph-convolutional neural nets. The resulting CGR GCNNs outperform other current approaches on a wide variety of data sets and prediction tasks. Furthermore, they perform well with a very limited model size, allowing for rapid training and evaluation. We could thus successfully extend the use of GCNNs from molecules to reactions, creating small and convenient models for the prediction of various reaction properties. We expect the developed representation and architecture, as well as the atom-mapped data sets made available along with this article, to seed further developments in the emerging field of reaction property prediction.

## Data and Software Availability

The CGR GCNN model architecture is available on GitHub on the master branch of Chemprop.^45^ Data sets 1, 3, and 8 are available from the literature,^20,46,52^ and were used as provided. Data sets 2, 4, 5, and 6 are available on GitHub.^53^ Data set 7 is proprietary and thus not freely available but does not provide an integral part of this study since it only complements data set 8. For all data sets except 7, we furthermore provide the data splits used in this study, as well as the trained CGR GCNN default models, along with instructions on how to create predictions.^53^

## Supporting Information Available

Figures showing MAE and RMSE of different models, analogous to Figures 3,4,5,7 and 8. Model performances on the Rad-6-RE database and detailed discussion of the influence of data leakage in this system. Details on hyperparameter searches and full list of test set performance (MAE, RMSE, and R^2^) for all models with and without hyperparameter optimization. (PDF)
