# Supplementary material for: Machine Learning of Reaction Properties via Learned Representations of the Condensed Graph of Reaction
Source: J Chem Inf Model. 2021 Nov 4;62(9):2101–10. doi: 10.1021/acs.jcim.1c00975 (PMC9092344; doi:10.1021/acs.jcim.1c00975)
Supplement: Supplementary file 1 — ci1c00975_si_001.pdf [file ci1c00975_si_001.pdf]

Supporting Information: Machine Learning of Reaction Properties via Learned Representations of the Condensed Graph of Reaction

Esther Heid and William H. Green*

Department of Chemical Engineering, Massachusetts Institute of Technology, Cambridge, Massachusetts 02139, United States

E-mail: whgreen@mit.edu

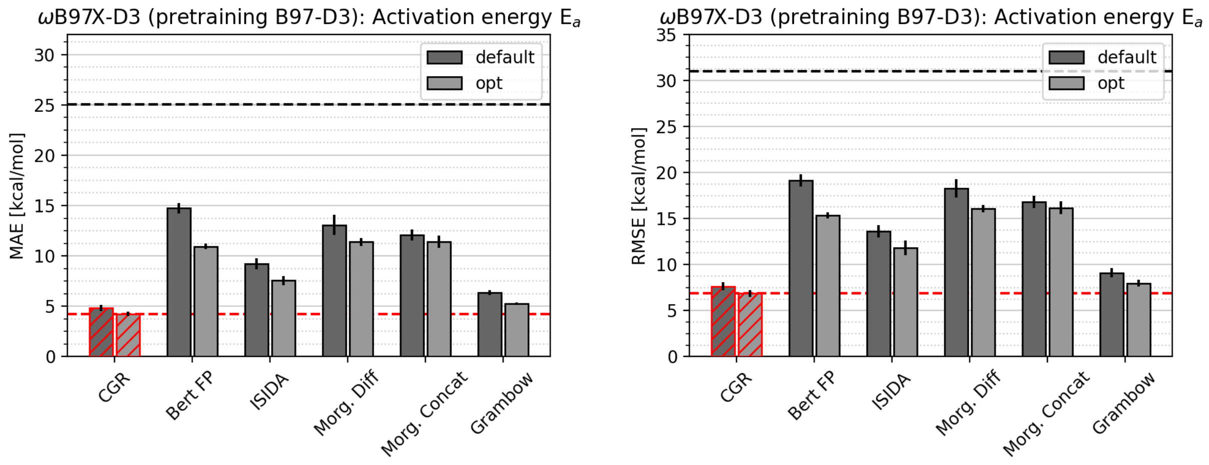

Figure S1: Comparison of test set mean absolute error (left) and root mean squared error (right) between different models for the ωB97X-D3 computational activation energy dataset, with pretraining on B97-D3 activation energies. Error bars correspond to the standard deviation between five folds. Dummy model performance indicated by dashed line and gray area (standard deviation between folds). Best model system highlighted in red, line corresponds to best performance.

S1 MAE and RMSE plots

Figures S1, S2, S3, S4, and S5 depict the MAEs and RMSEs across all systems, together with the performance of the dummy baseline model (mean: dashed black line, standard deviation between five folds: shaded area).

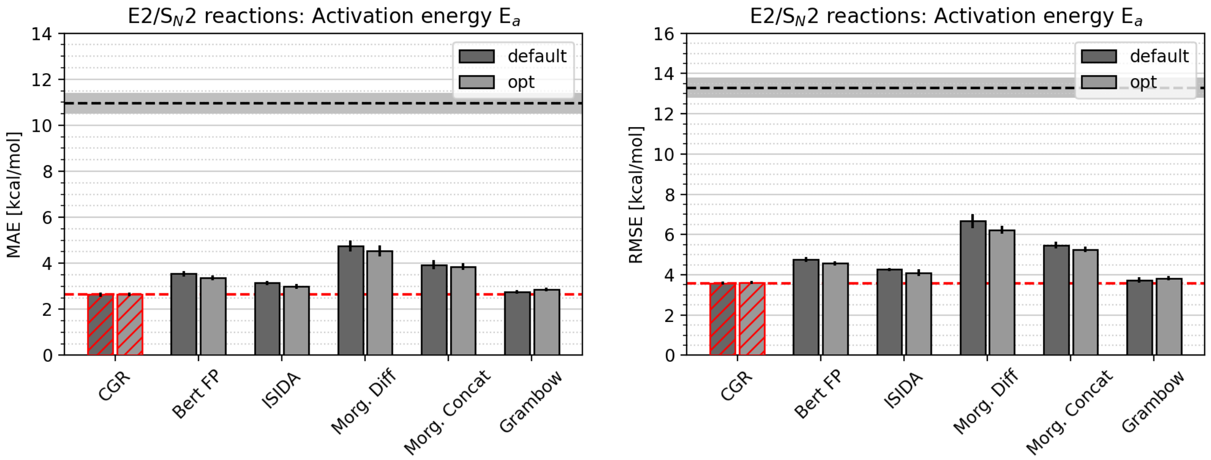

Figure S2: Comparison of test set mean absolute error (left) and root mean squared error (right) between different models for the E2/S$_N$2 computational activation energy dataset. Error bars correspond to the standard deviation between five folds. Dummy model performance indicated by dashed line and gray area (standard deviation between folds). Best model system highlighted in red, line corresponds to best performance.

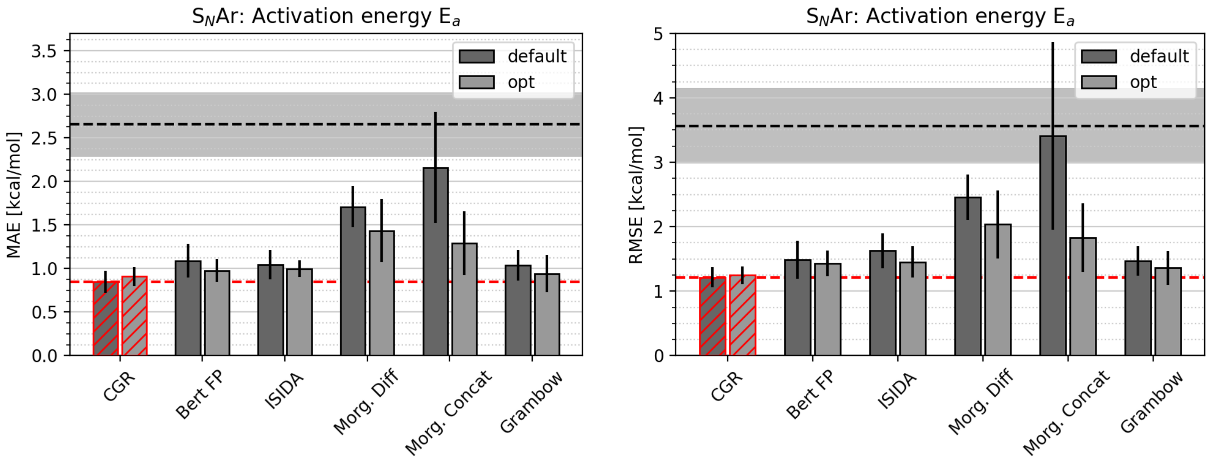

Figure S3: Comparison of test set mean absolute error (left) and root mean squared error (right) between different models for the S$_N$Ar experimental activation energy dataset. Error bars correspond to the standard deviation between five folds. Dummy model performance indicated by dashed line and gray area (standard deviation between folds). Best model system highlighted in red, line corresponds to best performance.

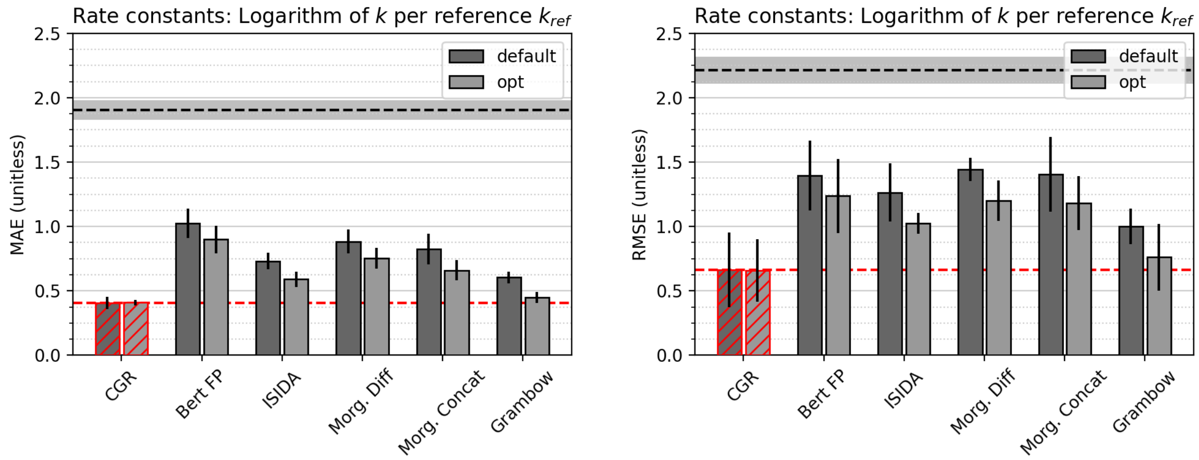

Figure S4: Comparison of test set mean absolute error (left) and root mean squared error (right) between different models for the computational rate constants dataset. Error bars correspond to the standard deviation between five folds. Dummy model performance indicated by dashed line and gray area (standard deviation between folds). Best model system highlighted in red, line corresponds to best performance.

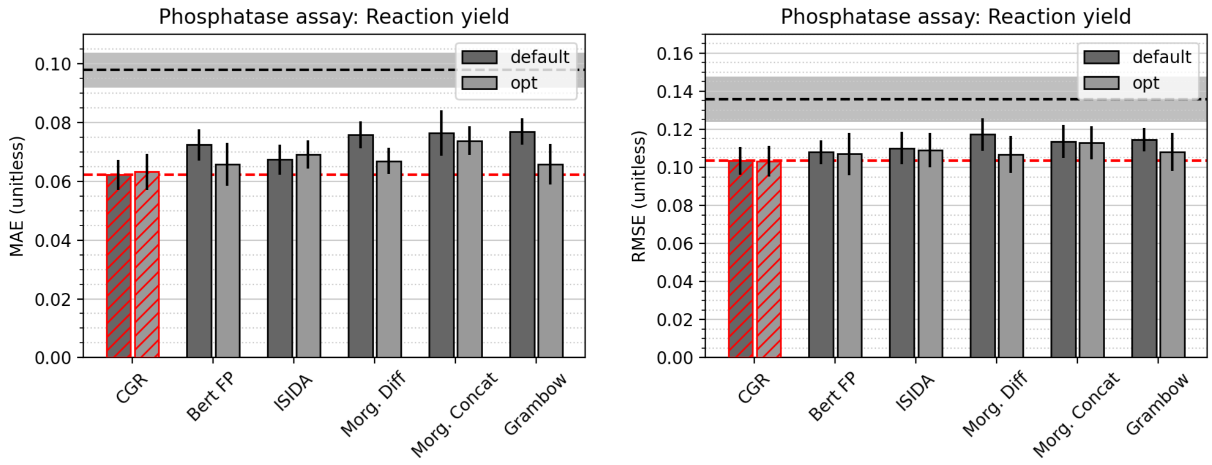

Figure S5: Comparison of test set mean absolute error (left) and root mean squared error (right) between different models for the xperimental phosphatase reaction yield dataset. Error bars correspond to the standard deviation between five folds. Dummy model performance indicated by dashed line and gray area (standard deviation between folds). Best model system highlighted in red, line corresponds to best performance.

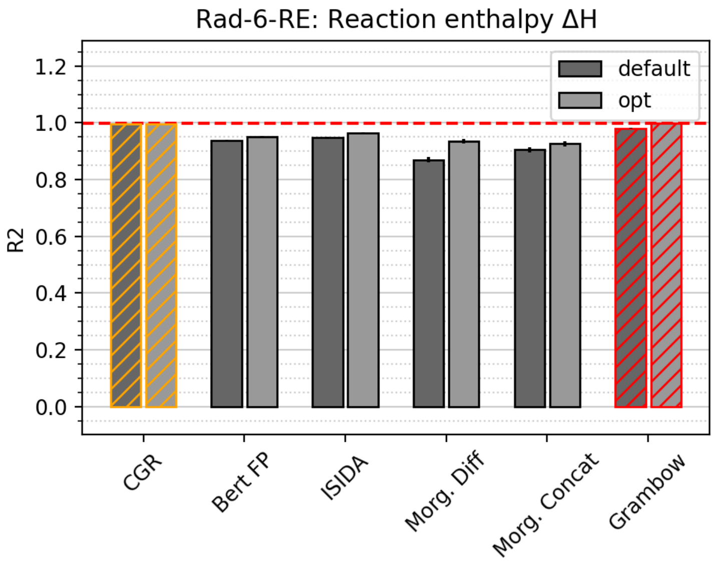

Figure S6: Comparison of test set R^2 scores between different models for the Rad-6-RE computational reaction enthalpy dataset. Error bars correspond to the standard deviation between five folds. Best model system highlighted in red or orange, line corresponds to best performance. Red: Best performance if Grambow's dual GCNN model allowed to relax to large numbers of FFN layer, which can learn the data by heart. Orange: Best performance if Grambow's dual GCNN model is constrained to one FFN layers, which reduces overfitting. The orange dot refers to the MAE of the dual GCNN in that case.

S2 Prediction of reaction enthalpies

Fig. S6 and S7 depict the respective model performances on the Rad-6-RE dataset. A performance comparison across different models is complicated, since the Rad-6-RE database consists of a reaction network, *i.e.* all possible reactions between a set of molecules. Is is therefore impossible to find a data split which separates out a completely independent test set without losing some of the data. The same problem was already described in Ref. S1. Since some of the molecules in the test sets thus also appear in the training sets, the true predictive accuracy of the models may be somewhat worse than the reported values, and the different models can exploit the data leakage to a different extent, as described in the following.

The CGR approach yields low mean absolute errors (0.13 eV), which come close to the errors reported in Ref. S1, although the model of Stocker *et al.* takes the geometries of each

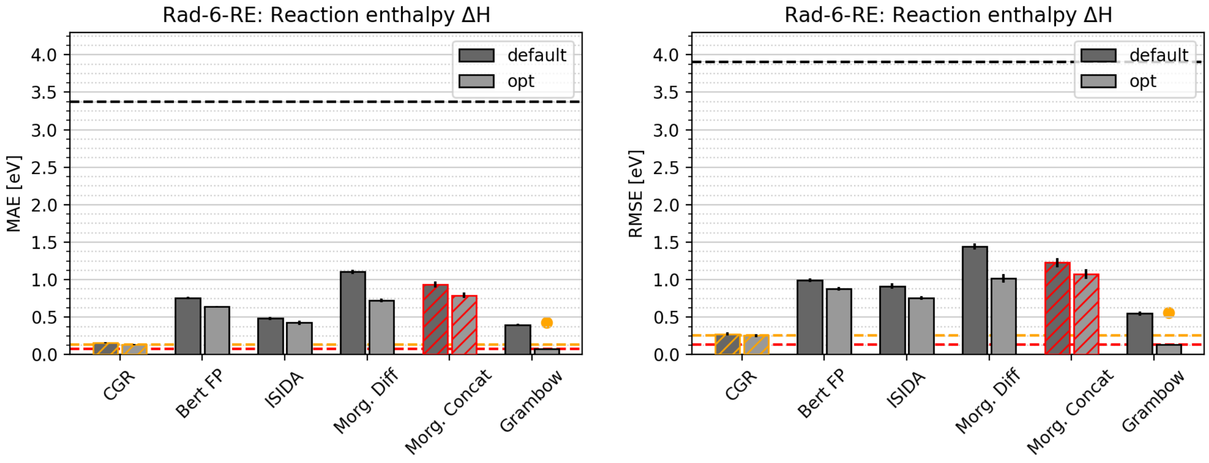

Figure S7: Comparison of test set mean absolute error (left) and root mean squared error (right) between different models for the Rad-6-RE computational reaction enthalpy dataset. Error bars correspond to the standard deviation between five folds. Dummy model performance indicated by dashed line. Best model system highlighted in red or orange, line corresponds to best performance. Red: Best performance if Grambow's dual GCNN model allowed to relax to large numbers of FFN layer, which can learn the data by heart. Orange: Best performance if Grambow's dual GCNN model is constrained to one FFN layers, which reduces overfitting. The orange dot refers to the MAE of the dual GCNN in that case.

molecule into account, thus leading to a more accurate representation of the enthalpy of a molecule. For force field geometries, their Kernel Ridge Regression (KRR) with the SOAP[S2] representation yields a mean absolute error of 0.09 eV, but if given DFT geometries as input the MAE is only 0.05 eV. The performance of the CGR GCNN method developed in this study, which does not require any 3-D information as input, is therefore very encouraging.

Grambow's dual GCNN model performs exceptionally well on this task, and outperforms the CGR model, leading to mean absolute errors of only 0.08 eV (red markers in Fig. S7). We attribute this performance boost to the architecture of the dual GCNN model, which subtracts the reactant embeddings from the product embeddings. Since the enthalpy of reaction itself is the subtraction of reactant enthalpies from the product enthalpies, this gives the dual GCNN model a large advantage for two reasons. First, the model can naturally pick up the structure of the data. Second, the model can exploit molecules which overlap between the training and test set. Good performances are only observed for a large number

of tunable paramaters, here 20M, and only at a large MPNN depth (which makes the molecular hidden representations unique) and with more than one FFN layer. This indicates that the model is in fact learning the energies of molecules by heart, instead of finding a meaningful relationship. The best performing model with a single FFN layer (orange markers in Fig. S7), where the model cannot easily learn the individual energies by heart, is above 0.4 eV. In contrast, the CGR GCNN model already yields acceptable performances at only 0.4M parameters and due to its architecture cannot exploit this data leakage, so that the CGR GCNN model still seems preferable.

Both GCNN approaches outperform fingerprint based models by a large margin.

S3 Model hyperparameters and performance

Table S1 and S2 list the hyperparameters, model sizes and performances for all systems and models. 'Opt' refers to the best parameters found by a hyperparameter search using 20 steps of Bayesian optimization as implemented in Chemprop,[S3] scanning MPNN depths of 2 to 6, 300 to 2400 hidden nodes in the MPNN and FFN, dropout rates of 0.0 to 0.4 and 1 (no hidden layer) to 3 FFN layers.

References

(S1) Stocker, S.; Csányi, G.; Reuter, K.; Margraf, J. T. Machine Learning in Chemical Reaction Space. *Nat. Commun.* **2020**, *11*, 1–11.

(S2) Bartók, A. P.; Kondor, R.; Csányi, G. On Representing Chemical Environments. *Phys. Rev. B* **2013**, *87*, 184115.

(S3) Yang, K.; Swanson, K.; Jin, W.; Coley, C.; Eiden, P.; Gao, H.; Guzman-Perez, A.; Hopper, T.; Kelley, B.; Mathea, M., et al. Analyzing Learned Molecular Representations for Property Prediction. *J. Chem. Inf. Model.* **2019**, *59*, 3370–3388.

Table S1: Number of parameters, hyperparameters (depth T, number of hidden neurons h, dropout d, number of FFN layers N) and performance for the activation energy datasets (E_a ωB97X-D3, E_a E2/S$_N$2 and E_a S$_N$Ar) of all models examined in this study. Intervals correspond to the mean and standard deviation of five folds.

Model	# Parameters	T	h	d	N	MAE	RMSE	Unit	R^2
E_a ωB97X-D3									
CGR default	378,601	3	300	0.0	2	4.84 ± 0.29	7.63 ± 0.43	kcal/mol	0.94 ± 0.01
CGR opt	2,817,101	6	1800	0.05	2	4.25 ± 0.19	6.88 ± 0.38	kcal/mol	0.95 ± 0.01
Grambow default	361,801	3	300	0.0	1	6.35 ± 0.26	9.11 ± 0.51	kcal/mol	0.92 ± 0.01
Grambow opt	24,877,601	4	2200	0.0	3	5.26 ± 0.15	7.98 ± 0.37	kcal/mol	0.94 ± 0.01
Bert default	77,401	-	300	0.0	2	14.73 ± 0.52	19.12 ± 0.66	kcal/mol	0.65 ± 0.02
Bert opt	6,381,601	-	2400	0.0	3	10.94 ± 0.29	15.34 ± 0.36	kcal/mol	0.77 ± 0.01
Morgan Diff default	307,801	-	300	0.0	2	13.08 ± 0.98	18.27 ± 0.98	kcal/mol	0.68 ± 0.03
Morgan Diff opt	8,224,801	-	2400	0.0	3	11.39 ± 0.39	16.10 ± 0.40	kcal/mol	0.75 ± 0.01
Morgan Concat default	615,001	-	300	0.0	2	12.08 ± 0.54	16.81 ± 0.69	kcal/mol	0.73 ± 0.02
Morgan Concat opt	10,682,401	-	2400	0.0	3	11.41 ± 0.58	16.16 ± 0.71	kcal/mol	0.75 ± 0.02
ISIDA default	13,159,801	-	300	0.0	2	9.21 ± 0.55	13.63 ± 0.66	kcal/mol	0.82 ± 0.02
ISIDA opt	72,747,201	-	1600	0.05	3	7.55 ± 0.48	11.80 ± 0.78	kcal/mol	0.87 ± 0.02
E_a E2/S$_N$2									
CGR default	378,601	3	300	0.0	2	2.64 ± 0.10	3.59 ± 0.09	kcal/mol	0.93 ± 0.01
CGR opt	2,817,101	6	1100	0.35	1	2.65 ± 0.09	3.61 ± 0.07	kcal/mol	0.93 ± 0.01
Grambow default	361,801	3	300	0.0	1	2.76 ± 0.08	3.74 ± 0.13	kcal/mol	0.92 ± 0.01
Grambow opt	16,754,401	6	1800	0.1	3	2.86 ± 0.07	3.83 ± 0.11	kcal/mol	0.92 ± 0.01
Bert default	77,401	-	300	0.0	2	3.54 ± 0.12	4.75 ± 0.12	kcal/mol	0.87 ± 0.01
Bert opt	361,201	-	1400	0.05	2	3.37 ± 0.10	4.56 ± 0.11	kcal/mol	0.88 ± 0.01
Morgan Diff default	307,801	-	300	0.0	2	4.75 ± 0.24	6.67 ± 0.35	kcal/mol	0.75 ± 0.02
Morgan Diff opt	7,652,101	-	2300	0.15	3	4.53 ± 0.23	6.24 ± 0.21	kcal/mol	0.78 ± 0.03
Morgan Concat default	615,001	-	300	0.0	2	3.94 ± 0.20	5.47 ± 0.17	kcal/mol	0.83 ± 0.02
Morgan Concat opt	2,665,001	-	1300	0.1	2	3.85 ± 0.15	5.25 ± 0.14	kcal/mol	0.84 ± 0.02
ISIDA default	55,801	-	300	0.0	2	3.14 ± 0.09	4.27 ± 0.07	kcal/mol	0.90 ± 0.01
ISIDA opt	334,801	-	1800	0.05	2	3.00 ± 0.10	4.10 ± 0.17	kcal/mol	0.90 ± 0.01
E_a S$_N$Ar									
CGR default	380,401	3	300	0.0	2	0.85 ± 0.12	1.22 ± 0.16	kcal/mol	0.87 ± 0.04
CGR opt	1,661,401	4	600	0.2	3	0.91 ± 0.11	1.25 ± 0.14	kcal/mol	0.87 ± 0.02
Grambow default	361,807	3	300	0.0	1	1.04 ± 0.17	1.46 ± 0.23	kcal/mol	0.82 ± 0.03
Grambow opt	8,278,201	5	1400	0.1	2	0.94 ± 0.21	1.36 ± 0.26	kcal/mol	0.84 ± 0.05
Bert default	79,201	-	300	0.0	2	1.09 ± 0.19	1.49 ± 0.29	kcal/mol	0.82 ± 0.03
Bert opt	6,396,001	-	2400	0.0	3	0.98 ± 0.13	1.43 ± 0.20	kcal/mol	0.83 ± 0.06
Morgan Diff default	309,601	-	300	0.0	2	1.71 ± 0.24	2.46 ± 0.35	kcal/mol	0.50 ± 0.10
Morgan Diff opt	1,341,601	-	1300	0.1	2	1.44 ± 0.36	2.04 ± 0.53	kcal/mol	0.67 ± 0.08
Morgan Concat default	616,801	-	300	0.0	2	2.16 ± 0.64	3.41 ± 1.46	kcal/mol	0.05 ± 0.50
Morgan Concat opt	2,672,801	-	1300	0.4	2	1.29 ± 0.37	1.83 ± 0.54	kcal/mol	0.73 ± 0.08
ISIDA default	69,301	-	300	0.0	2	1.05 ± 0.17	1.63 ± 0.27	kcal/mol	0.76 ± 0.12
ISIDA opt	6,316,801	-	2400	0.0	3	1.00 ± 0.10	1.45 ± 0.24	kcal/mol	0.81 ± 0.09

Table S2: Number of parameters, hyperparameters (depth T, number of hidden neurons h, dropout d, number of FFN layers N) and performance for the enthalpy, kinetics and yield datasets (ΔH Rad-6-RE, $log(k)$ Reaction rates and Yield Phosphatases) of all models examined in this study. Intervals correspond to the mean and standard deviation of five folds.

Model	# Parameters	T	h	d	N	MAE	RMSE	Unit	R^2
ΔH Rad-6-RE									
CGR default	378,601	3	300	0.0	2	0.16 ± 0.01	0.28 ± 0.02	eV	1.00 ± 0.00
CGR opt	10,371,601	6	1800	0.05	2	0.13 ± 0.01	0.25 ± 0.02	eV	1.00 ± 0.00
Grambow default	361,801	3	300	0.0	1	0.40 ± 0.01	0.55 ± 0.03	eV	0.98 ± 0.00
Grambow opt	20,035,401	5	2200	0.0	2	0.08± 0.00	0.14 ± 0.01	eV	1.00 ± 0.00
Bert default	77,401	-	300	0.0	2	0.76 ± 0.02	0.99 ± 0.03	eV	0.94 ± 0.00
Bert opt	6,381,601	-	2400	0.0	3	0.65 ± 0.01	0.88 ± 0.02	eV	0.95 ± 0.00
Morgan Diff default	307,801	-	300	0.0	2	1.11 ± 0.03	1.44 ± 0.04	eV	0.87 ± 0.01
Morgan Diff opt	8,224,801	-	2400	0.0	3	0.72 ± 0.02	1.02 ± 0.06	eV	0.94 ± 0.01
Morgan Concat def.	615,001	-	300	0.0	2	0.94 ± 0.04	1.23 ± 0.06	eV	0.90 ± 0.01
Morgan Concat opt	10,682,401	-	2400	0.0	3	0.79 ± 0.04	1.08 ± 0.07	eV	0.93 ± 0.01
ISIDA default	4,291,501	-	300	0.0	2	0.48 ± 0.02	0.92± 0.04	eV	0.95 ± 0.00
ISIDA opt	8,943,601	-	600	0.1	3	0.43 ± 0.03	0.76 ± 0.02	eV	0.96± 0.00
$log(k)$ Reaction rates									
CGR default	378,601	3	300	0.0	2	0.41 ± 0.05	0.66 ± 0.29		0.90 ± 0.09
CGR opt	6,393,701	5	1700	0.25	1	0.41 ± 0.02	0.66 ± 0.24		0.90 ± 0.07
Grambow default	361,801	3	300	0.0	1	0.60 ± 0.05	1.00 ± 0.14		0.79 ± 0.05
Grambow opt	8,269,801	6	1400	0.1	2	0.45 ± 0.04	0.76 ± 0.26		0.87 ± 0.08
Bert default	77,401	-	300	0.0	2	1.03 ± 0.11	1.40 ± 0.27		0.59 ± 0.14
Bert opt	6,381,601	-	2400	0.0	3	0.90 ± 0.11	1.24 ± 0.29		0.67 ± 0.14
Morgan Diff default	307,801	-	300	0.0	2	0.88 ± 0.09	1.44 ± 0.09		0.56 ± 0.04
Morgan Diff opt	8,224,801	-	2400	0.0	3	0.75 ± 0.08	1.20 ± 0.16		0.70 ± 0.07
Morgan Concat def.	615,001	-	300	0.0	2	0.83 ± 0.12	1.41 ± 0.29		0.58 ± 0.15
Morgan Concat opt	2,049	-	-	0.05	1	0.66 ± 0.08	1.18 ± 0.21		0.71 ± 0.08
ISIDA default	200,101	-	300	0.0	2	0.73 ± 0.06	1.26 ± 0.23		0.79 ± 0.05
ISIDA opt	7,363,201	-	2400	0.0	3	0.59 ± 0.06	1.03 ± 0.08		0.87 ± 0.08
Yield Phosphatases									
CGR default	444,001	3	300	0.0	2	0.062 ± 0.005	0.103 ± 0.007		0.41 ± 0.08
CGR opt	6,692,001	3	1400	0.05	2	0.063 ± 0.006	0.103 ± 0.008		0.41 ± 0.07
Grambow default	362,019	3	300	0.0	1	0.077 ± 0.004	0.115 ± 0.006		0.27 ± 0.07
Grambow opt	6,390,001	4	1200	0.05	2	0.066 ± 0.007	0.108 ± 0.010		0.35 ± 0.12
Bert default	142,801	-	300	0.0	2	0.072 ± 0.005	0.108 ± 0.006		0.35 ± 0.09
Bert opt	6,387,101	-	2300	0.15	3	0.066 ± 0.007	0.107 ± 0.011		0.37 ± 0.07
Morgan Diff default	373,201	-	300	0.0	2	0.076 ± 0.005	0.117 ± 0.009		0.23 ± 0.14
Morgan Diff opt	1,107,001	-	600	0.2	3	0.067 ± 0.005	0.107 ± 0.010		0.37 ± 0.08
Morgan Concat def.	680,401	-	300	0.0	2	0.076 ± 0.008	0.113 ± 0.009		0.28 ± 0.15
Morgan Concat opt	2,948,401	-	1300	0.1	2	0.074 ± 0.005	0.113 ± 0.009		0.29 ± 0.13
ISIDA default	81,301	-	300	0.0	2	0.067 ± 0.005	0.110 ± 0.009		0.32 ± 0.12
ISIDA opt	487,801	-	1800	0.05	2	0.069 ± 0.005	0.107 ± 0.10		0.37 ± 0.08
